# Asymptomatic infection with highly pathogenic avian influenza H5N1 in wild birds: how sound is the evidence?

**DOI:** 10.1186/1743-422X-3-96

**Published:** 2006-11-17

**Authors:** Chris J Feare, Maï Yasué

**Affiliations:** 1 WildWings Bird Management, 2 North View Cottages, Grayswood Common, Haslemere, Surrey GU27 2DN, UK; 2 BirdLife International, Wellbrook Court, Girton Road, Cambridge CB3 0NA, UK

## Abstract

**Background:**

Widespread deaths of wild birds from which highly pathogenic avian influenza virus H5N1 has been isolated suggest that the virus continues to be lethal to them. However, asymptomatic carriage by some wild birds could allow birds to spread the virus on migration. Confirmation of such carriage is therefore important for the design of mitigation measures for the disease in poultry.

**Discussion:**

Two recent papers have reported the isolation of H5N1 from a small number of water birds in China and Russia and have concluded that wild birds can spread the viruses over long distances on migration. However, both papers contain weaknesses in the provision of ornithological and associated data that compromise conclusions that can be reached about the role of wild birds in the spread of H5N1. We describe the weaknesses of these studies and highlight the need for improved methodological description and methodology, where appropriate, and further research.

**Summary:**

A rigorous assessment of whether wild birds can carry H5N1 asymptomatically is critical to evaluating the risks of spread by migratory birds on long-distance migration.

## Background

Infected dead or sick wild birds have occasionally been found since the beginning of the current outbreak of highly pathogenic avian influenza (HPAI) H5N1 in southern China, in 1997. In 2005, however, a large number of migrant aquatic birds died at Lake Qinghai in north-western China, and were found to be infected with HPAI H5N1 [1-3]. Since then, dead or sick water birds, and some other species, have been recorded more frequently in Asia and Europe. This has led to the notion that migrating wild birds could have spread the virus from east Asia, across southern Siberia, and into Europe and Africa in 2005 and 2006 [4]. A key question relating to this hypothesis is whether some wild birds are infected with HPAI H5N1 asymptomatically. If some infected wild birds show few symptoms and are not incapacitated, they may be able to fly long distances while shedding the virus and contribute significantly to the global spread of H5N1 during the autumn or spring migration. This has obvious implications for the emphasis of H5N1 mitigation measures or risk analysis.

Asymptomatic infection with Asian lineage HPAI H5N1 has been reported in several species of experimentally infected laboratory waterfowl [5], and also in poultry in live bird markets [6]. In wild birds, the only convincing evidence of asymptomatic infection is from non-migratory tree sparrows *Passer montanus *in Henan province, China [7], but the genotype of this HPAI H5N1 was different from that responsible for outbreaks in poultry and some wild birds in Asia, and now into Europe and Africa. At present the evidence for asymptomatic infection in wild migratory birds is based on only two scientific papers, both of which have weaknesses in the ornithological data supplied. The aim of this paper is to demonstrate why both these studies failed to demonstrate conclusively asymptomatic infection in wild migratory birds and to highlight the data requirements to assess whether healthy wild birds can carry the virus and spread it over long distances along migration routes.

## Discussion

Chen et al. [8] recently reported the isolation of HPAI H5N1 viruses from six apparently healthy wild migratory birds at Poyang Lake, Jiangxi province, China, in January and March 2005 and concluded that their finding indicated that wild birds are able to disseminate the virus over long distances. Similarly, Lvov et al. [9] reported finding HPAI H5N1 in clinically healthy wild ducks (mallards *Anas platyrhynchus *and pochard *Aythya ferina*) and in another water bird, great-crested grebe *Podiceps cristatus*, at Lake Chany, Novosibirsk, Russia, during an outbreak in poultry. They used this evidence to support their claim that poultry had become infected with the virus as a result of its circulation in wild birds. However, poor methodological description of the field sampling of wild birds, or poor methodology, in both of these papers cast doubt on the interpretation that these wild birds were carrying the virus asymptomatically. There are two areas of concern: failure to accurately identify the species of wild duck from which isolates were obtained, and failure to report how wild birds were procured for sampling. Our aim here is to highlight these difficulties in presentation or in data collection in the hope of avoiding these problems in future avian influenza surveillance efforts.

### Specific identity of birds sampled

While Lvov et al. [9] identified their sampled birds to species, Chen *et al*. [8] only identified the birds from which they isolated two genotypes of HPAI H5N1, as "migratory ducks". The authors state that three species of "migratory duck" were sampled at the lake – mallard *Anas platyrhynchos*, falcated teal *Anas falcata *and spot-billed duck *Anas poecilorhyncha*. Of these, falcated teal are migratory, but while most mallard in this part of China are considered to be migratory, some are believed to breed at Poyang Lake (Mark Barter in litt.). Spot-billed ducks, which breed over most of China [10], including at Poyang [11], are the commonest breeding duck at the lake (Li Fengshan, pers. comm.). Many spot-billed ducks at Poyang Lake in winter are thus likely to be non-migratory, but morphologically indistinguishable from those that do migrate. Additionally, local villagers release many domestic ducks on to the lake and its margins and these may share habitats with wild ducks (J. Burnham, pers. comm.). Most domestic ducks are descended from mallards and many domestic forms resemble their progenitors. Without careful identification there is thus a risk that domestic ducks might have been sampled.

Unless the precise identity of captured birds from which samples have been taken is known, categorisation into migratory and non-migratory, or even wild, is not possible. Poor identification of wild birds appears commonplace in reports of H5N1. For example, disease outbreak reports from the World Organisation for Animal Health [12] frequently refer to "wild bird", "wild duck", "pigeon", "gull" etc. Such imprecise identification limits our understanding of the possible role of wild birds in H5N1 epidemiology and thus represents a waste of potentially valuable information. Moreover identifying the species that are capable of carrying H5N1 asymptomatically is important so that H5N1 mitigation/response efforts can focus on reducing contact between poultry and these key species.

### Capture methods for wild birds

Neither Lvov et al. [9] nor Chen et al [8] described how the birds that they sampled were caught. Catch method should be described because the methods used may select for individuals of differing health status. For example, catching birds using bait can bias samples toward hungry or young individuals with reduced body condition, or birds that are sick. Birds that have been caught using hand nets must be highly tolerant of man, and thus possibly domesticated or sick. Hunting with shot guns restricts sampling to birds that are within close range, again raising the possibility of selecting for individuals more tolerant of man or less able to escape. Hunting with rifles can target birds at a greater distance, but the requirement for greater precision when aiming may select for less active individuals, which again could be sick. Most sampling methods have inherent biases in the individuals that are most likely to be caught [13] and therefore the method of sample acquisition should be described and the possible effects on health status of sampled individuals discussed.

Furthermore, although both the Lvov and Chen studies described the birds as clinically healthy, the basis of this conclusion was not given. They did not give the sex or age of the birds, their body masses, stage of moult, plumage condition or other indicators of body condition, leaving doubt as to their health status.

### Asymptomatic infection in migrating birds

In addition to following the above guidelines and successfully demonstrating that a *healthy *individual of a *migratory species *carried H5N1, in order to show that wild birds can play a major role in the global spread of the disease, positive test results should be obtained during the migration seasons.

The birds at Poyang Lake were captured in mid and late winter, while those at Lake Chany were obtained in July, towards the end of the annual moult when ducks and grebes shed their primary feathers simultaneously and become temporarily flightless. Importantly, neither group of birds was sampled at the time of seasonal migration. Even if birds may be able to carry H5N1 asymptomatically during the winter or moulting season this does not necessarily imply that birds may be able to carry H5N1 during migration. Long-distance migration imposes energetic and physiological stress, which has been shown in waders to reduce immunocompetence [14]. Similar studies have yet to be undertaken in migrating ducks, which often undertake shorter flights between resting areas than waders [15,16]. However, ducks have a much higher wing-loading than waders [17], and thus their shorter flights may nevertheless incur high flight costs and impose significant stresses.

The evidence from most wild bird outbreaks to date, which have involved dead or dying birds, suggests that the virus remains highly lethal to them and if the immune systems of migrating ducks are impaired, their ability to carry a virulent pathogen over long distances may well be compromised. The largest well-documented HPAI H5N1 wild bird outbreak occurred at Lake Qinghai in 2005 [3], several weeks after migratory birds arrived at the lake, strongly suggesting that the virus was acquired at the destination [18], rather than carrying it during migration.

The incursion of the virus into Europe in spring 2006 affected birds, especially mute swans *Cygnus olor*, apparently during dispersal away from freezing conditions in the Black/Caspian sea areas, but not on long-distance seasonal migration. The mechanism of virus transmission during this event remains unknown, but could have involved infected birds travelling short distances before death, or as yet unknown asymptomatic carriers. Better quality data collection on the European wild bird outbreaks may have helped to assess the possibility of asymptomatic carriage by some birds, identify the mechanism of transmission and contribute to appropriate mitigation strategies.

In conclusion, the two papers discussed here [8,9] lack details that provide convincing evidence that the birds they sampled were carrying HPAI H5N1 asymptomatically and thus that they could have spread the virus during long-distance migration. Without clear information on the identity of the species sampled, the methods used to catch birds, or methods used to assess health in birds, evidence for asymptomatic transmission of HPAI H5N1 viruses in wild birds will remain circumstantial. There is a clear need for more and better data on the potential for some wild birds to carry the virus asymptomatically, especially on migration, and in captive wild species subjected to the kinds of stresses that they would experience during migration. These types of studies will provide a more definitive assessment of the extent to which wild birds are capable of transmitting HPAI H5N1 along migration routes, and thus provide information valuable for the construction of risk analyses and the development and location of appropriate biosecurity measures.

## Summary

Two research papers have claimed to have isolated HPAI H5N1 from apparently healthy wild birds, implicating them as agents in the spread of the virus over long distances on migration. We highlight weaknesses in the description of the methodology, or in the methodology itself, of sampling wild birds which cast doubt on the asymptomatic carriage of the virus by healthy birds. Ways in which the collection of samples of wild birds can be improved in studies of asymptomatic carriage of HPAI H5N1 are given.

## Abbreviations

HPAI: highly pathogenic avian influenza.

## Competing interests

The author(s) declare that they have no competing interests.

## Authors' contributions

CJF and MY jointly wrote the manuscript and approved the final version.

## References

[B1] Chen H, Smith GJD, Zhang SY, Qin K, Wang J, Li KS, Webster RG, Peiris JSM, Guan Y (2005). H5N1 virus outbreak in migratory waterfowl. Nature.

[B2] Liu J, Xiao H, Lei F, Zhu Q, Qin K, Zhang XW, Zhaing XI, Dhao D, Wang G, Feng Y, Ma J, Liu W, Wang J, Gao F (2005). Highly pathogenic H5N1 influenza virus infection in migratory birds. Science.

[B3] Chen H, Li Y, Li Z, Shi J, Shinya K, Deng G, Qi Q, Tian G, Fan S, Zhao H, Sun Y, Kawaoka Y (2006). Properties and dissemination of H5N1 viruses isolated during and influenza outbreak in migratory waterfowl in western China. J Virol.

[B4] Normile D (2006). Evidence points to migratory birds in H5N1 spread. Science.

[B5] Sturm-Ramirez KM, Hulse-Post DJ, Govorkova EA, Humberd J, Seiler P, Puthavathana P, Buranathai C, Nguyen TD, Chaisingh A, Long HT, Naipospos TSP, Chen H, Ellis TM, Guan Y, Peiris JSM, Webster RG (2005). Are ducks contributing to the endemicity of highly pathogenic H5N1 influenza virus in Asia?. J Virol.

[B6] Nguyen DC, Uyeki TM, Jadhao S, Maines T, Shaw M, Matsuoka Y, Smith C, Rowe T, Lu X, Hall H, Xiyan X, Balish A, Klimov A, Tumpey TM, Swayne DE, Huynh LPT, Nghiem HK, Nguyen HHT, Hoang LT, Cox NJ (2005). Isolation and characterization of avian influenza viruses, including highly pathogenic H5N1, from poultry in live bird markets in Hanoi, Vietnam, in 2001. J Virol.

[B7] Kou Z, Lei FM, Yu J, Fan ZJ, Yin ZH, Jia CX, Xiong KJ, Sun YH, Zhang XW, Wu XM, Gao XB, Lil TX (2005). New Genotype of Avian Influenza H5N1 Viruses Isolated from Tree Sparrows in China. J Virol.

[B8] Chen H, Li KS, Wang J, Fan XH, Rayner JM, Vijaykrishna D, Zhang JX, Zhang LJ, Guo CT, Cheung CL, Xu KM, Duan L, Huang K, Qin K, Leung YHC, Wu WL, Lu HR, Chen Y, Xia NS, Naipospos TSP, Yuen KY, Hassan SS, Bahri S, Nguyen TD, Webster RG, Peiris JSM, Guan Y (2006). Establishment of multiple sublineages of H5N1 influenza virus in Asia: Implications for pandemic control. Proc Nat Acad Sci.

[B9] Lvov DK, Schelkanov MIU, Deriabin PG, Grebennikova TV, Prilipov AG, Nepoklonov Ye A, Onishchenko GG, Vlasov NA, Aliper TI, Zaberezhnyi AD, Kireyev Ye D, Krasheninnikov OP, Kiryukhin ST, Burtseva Ye I, Slepushkin AN (2006). Isolation of influenza A/H5N1 virus strains from poultry and wild birds in west Siberia during epizooty (July 2005) and their depositing to the state collection of viruses (August 2005). [In Russian]. Vopr Virusol.

[B10] Madge S, Burn H (1988). Wildfowl An identification guide to the ducks, geese and swans of the world.

[B11] Cheng T-H (1976). Distributional list of Chinese birds.

[B12] OIE Update on avian influenza in animals (Type H5). http://www.oie.int.

[B13] Aebischer NJ (1986). Estimating the proportion of uncatchable animals in a population by double-sampling. Biometrics.

[B14] Guglielmo CG, Piersma T, Williams TD (2001). A sport-physiological perspective on bird migration: evidence for flight-induced muscle damage. J Ex Biol.

[B15] Veen J, Yurlov AK, Delany SN, Mihantiev AI, Selivanova MA, Boere GC (2005). An atlas of movements of southwest Siberian waterbirds.

[B16] Bairlein F, Wernham C, Toms M, Marchant J, Clark J, Siriwardina G, Baillie S (2002). How do birds migrate? The behavioural physiology of migration and orientation. The migration atlas.

[B17] McNeill Alexander R, Brooke M, Birkhead T (1991). Movement. The Cambridge encyclopedia of ornithology.

[B18] Feare CJ The role of wild birds in the spread of HPAI H5N1. Avian Dis.

